# Gallium arsenide waveguides as a platform for direct mid-infrared vibrational spectroscopy

**DOI:** 10.1007/s00216-020-02546-3

**Published:** 2020-03-31

**Authors:** Julian Haas, Robert Stach, Claudia Kolm, Rudolf Krska, Boris Mizaikoff

**Affiliations:** 1 Institute of Analytical and Bioanalytical Chemistry, Ulm University, Albert-Einstein-Allee 11, 89081 Ulm, Germany; 2 Institute of Bioanalytics and Agro–Metabolomics, Department of Agrobiotechnology (IFA–Tulln), University of Natural Resources and Life Sciences Vienna (BOKU), Konrad Lorenzstr. 20, 3430 Tulln, Austria; 3 Institute for Global Food Security, School of Biological Sciences, Queens University Belfast, University Road, Belfast, Northern Ireland BT7 1NN UK

**Keywords:** Gallium arsenide, Surface modification, Evanescent field absorption, Self-assembled monolayers, Surface-enhanced infrared absorption, Mid-infrared chem/biosensor

## Abstract

During recent years, mid-infrared (MIR) spectroscopy has matured into a versatile and powerful sensing tool for a wide variety of analytical sensing tasks. Attenuated total reflection (ATR) techniques have gained increased interest due to their potential to perform non-destructive sensing tasks close to real time. In ATR, the essential component is the sampling interface, i.e., the ATR waveguide and its material properties interfacing the sample with the evanescent field ensuring efficient photon-molecule interaction. Gallium arsenide (GaAs) is a versatile alternative material vs. commonly used ATR waveguide materials including but not limited to silicon, zinc selenide, and diamond. GaAs-based internal reflection elements (IREs) are a new generation of semiconductor-based waveguides and are herein used for the first time in direct spectroscopic applications combined with conventional Fourier transform infrared (FT-IR) spectroscopy. Next to the characterization of the ATR waveguide, exemplary surface reactions were monitored, and trace-level analyte detection via signal amplification taking advantage of surface-enhanced infrared absorption (SEIRA) effects was demonstrated. As an example of real-world relevance, the mycotoxin aflatoxin B1 (AFB1) was used as a model analyte in food and feed safety analysis.

**Figure:**
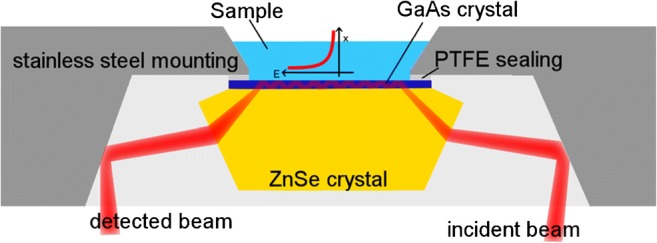
Graphical abstract

Graphical abstract

## Introduction

Spectroscopy in the mid-infrared spectral region (MIR, 2.5–25 μm) has nowadays evolved into a routinely applied analytical tool. Inherently non-destructive analysis as well as well-pronounced fundamental rotational, vibrational, and roto-vibrational molecular transitions in the MIR renders this technique suitable for a wide variety of analytical sensing tasks. Especially, attenuated total reflection (ATR) techniques have become increasingly popular for probing samples opaque in the MIR such as (bio)medical specimen within aqueous matrices. ATR sensing is based on the fundamental phenomenon of total internal reflection within a high-refractive-index waveguide material. Upon reflection at the analyte/internal reflection element (IRE) interface, an intensity exponentially decaying evanescent field is reaching into the adjacent media, which allows analyte interaction (e.g., via absorption) and the generation of ATR spectra. The penetration depth *d*_p_ of the evanescent field reaches to a distance of approx. 1/10 of the deployed wavelength *λ* into the adjacent media, as described via Eq. (1).

1 $$ {d}_{\mathrm{p}}=\frac{\lambda }{2\pi {n}_1\sqrt{\sin^2\left(\theta \right)-{\left(\frac{n_2}{n_1}\right)}^2}} $$

As evident from Eq. (1), the refractive indices of the ATR waveguide *n*_1_ and the sample *n*_2_ along with the angle of incidence *θ* are critical parameters, which are determining the sample interaction volume and the related detection capabilities besides the deployed wavelength. However, a limited set of IRE materials are suitable for ATR sensing in the MIR [1]. Among the routinely used IRE waveguide materials are silicon (Si), zinc selenide (ZnSe), germanium (Ge), and diamond, whereas selection of the most suitable IRE waveguide is dependent on the anticipated field of application. In the present study, we demonstrate the utility of gallium arsenide (GaAs) as a promising alternative in the context of biorecognition sensing schemes. III/V semiconductor materials such as GaAs are well known in electronic circuit design for high-frequency data transfer, and are readily structured after epitaxial growth via wet and dry etching protocols. For photonic sensing devices, different structural sensing architectures based on thin-film GaAs waveguide technology have been recently pioneered by the research team of Mizaikoff and collaborators. However, to date, such sensing concepts including slab waveguides [2–4] and Mach-Zehnder interferometers (MZI) have been combined with cascade laser light sources (i.e., quantum and interband cascade lasers [QCLs, ICLs]) rather than broadband infrared spectrometers [5–7]. Hence, the present study for the first time combines GaAs waveguides with Fourier transform infrared (FT-IR) spectroscopy facilitating a more ubiquitous usage of this new generation of waveguide materials.

Waveguide-based sensor devices may in addition benefit from surface modification strategies for enhancing the sensor response leading to improved limits of detection (LODs). Exemplarily, analyte enrichment at the sensor surface and within the penetration depth of the evanescent field may be performed via chemical strategies such as preconcentration membranes, or biorecognition approaches such as immobilized enzymes, antibodies, or aptamers. In addition, the sensor robustness may be improved by protecting the IRE surface against, e.g., chemical degradation with self-assembled monolayers (SAMs) as routinely employed architectural motive [8]. Thiol-terminated long-chained organic molecules are in fact ideally suited for GaAs surface modification, as—similarly to gold—thiol anchoring groups also strongly bind to GaAs [9, 10]. Bifunctional SAMs allow for further chemical modification after attachment of a first monolayer [5, 11]. It should be noted though that passivating native oxide layers have to be removed from the GaAs surface prior to any immobilization scheme [12].

Due to similar binding chemistries, the immobilization of gold nanoparticles (AuNPs) at the GaAs waveguide surface is facilitated enabling additional amplification of the analytical signal via surface-enhanced infrared absorption (SEIRA) spectroscopy [13, 14]. Thereby, signal amplification in ATR-based sensors for probing, e.g., small molecules such as p-nitro benzoic acid or biologically relevant molecules including DNA or membrane proteins has been demonstrated [15–23]. Commonly, spherical nanoparticle structures are used in surface-enhanced spectroscopies. More recently, alternative geometries including plasmonic nanostars have demonstrated superior signal amplification in MIR sensing scenarios [24].

Given the anticipated increase in sensitivity, the present study has focused on the potential detection of mycotoxins, which have to be reliably detected in feed and food matrices such as corn, rice, maize, and milk at ppm-ppb concentration levels, as mandated by the respective authorities [25–27]. As a relevant example of such secondary fungus metabolites, aflatoxin B1 (AFB1) has been selected, which is considered among the most carcinogenic herbal substances known [28]. Conventionally, analytical techniques based on HPLC, LCMS, or GC/MS-MS are utilized for molecular discrimination of food pathogens [29]. However, such methods are time and cost consuming and are usually limited in portability. Alternatively, MIR-based sensing systems are promising device platforms for monitoring scenarios, albeit not competitive in molecular discrimination and sensitivity vs. the formerly mentioned laboratory techniques. In addition, MIR-based sensors may be significantly reduced in size towards highly integrated portable devices utilizing integrated photonics with a common material system such as GaAs for light source, detector, and waveguide sensing interface. While the direct detection of mycotoxins may be limited with ATR-based IR sensors [30–32], the evaluation of matrix changes resulting from fungal activity after infection during growth, harvest, or storage scenarios may be promising. As previously shown, matrix changes are indeed correlated with the presence of mycotoxins, as shown via FT-IR- and QCL-based techniques with sensitivities at EU regulatory limits [33–38]. However, utilizing broadband spectra of selected mycotoxins along with smart extraction schemes may increase the utility of MIR monitoring systems, as shown for the example of AFB1.

## Experimental section

### Chemicals and materials

Sodium acetate (C_2_H_3_NaO_2_, water free), gold (III) chloride trihydrate (HAuCl_4_ × 3 H_2_O), trisodium citrate dihydrate (C_6_H_5_O_7_Na_3_ × 2 H_2_O), concentrated hydrochloric acid (HCl, 32%), l(+)-ascorbic acid (AA), silver nitrate (AgNO_3_), and sodium hypochlorite (NaClO, 14% Cl_2_ in aqueous solution) were purchased from Sigma-Aldrich. HS-PEG-SH (MW, 2000) was purchased from Rapp Polymere GmbH (Tübingen, Germany). Aflatoxin B1 was purchased from Acros Organics (NJ, USA). Absolute ethanol (EtOH), methanol (MeOH), chloroform (CHCl_3_), acetonitrile (ACN), acetone (Propan-2-one), and isopropyl alcohol (IPA) were purchased from VWR. Milli-Q water (10.8 MΩ at 21 °C, Millipore) was used in all experiments.

Commercially available diamond ATR crystals were purchased from Harrick Scientific (Harrick Scientific Products Inc., Pleasantville, NY, USA) and used without any further treatment. Si ATR crystals were laser cut from 3-in. wafers (crystal orientation <100>, thickness 380 ± 2 μm, prime FZ, undoped, double side polished (DSP), TTV < 10 μm, resistivity 10 k–100 kΩ cm; Microchemicals GmbH, Ulm, Germany). Debris from the cutting process was removed via subsequent sonication in acetone, IPA, and water. Further cleaning was performed according the RCA 1 and RCA 2 protocols to remove possible metal contaminations and to degrease the surface [39, 40]. Furthermore, this treatment ensured a uniform oxide layer on the silicon crystal surface. GaAs ATR crystals were laser-cut from 2-in. wafers (crystal orientation <100>, thickness 300 μm, undoped, DSP, resistivity 2 MΩ cm; AXT Europe, GEO Semiconductor Ltd., Geneva, Switzerland). Debris from the cutting process was removed as for the silicon crystals. Subsequent immersion in concentrated HCl (32%) and rinsing in water with subsequent drying in a nitrogen stream further removed residual debris and ensured growth of a thin and uniform native oxide layer on the GaAs crystal surface.

Basic material characteristics of the utilized ATR crystals are summarized in Table 1. Apart from the optical properties, such as the refractive index and the utilizable wavelength range, physical resilience properties additionally affect usage of the individual waveguide materials. As diamond is predominant for sensing in harsh and abrasive scenarios, suitable waveguides for chem/biosensing of aqueous samples are mainly governed by the accessible pH range.

**Table 1:** Material properties of the ATR waveguide materials investigated in the present study

Material	Wafer disk thickness (μm)	Angle of incidence (°)	Internal reflections	Refractive index *n* at 1000 cm^−1^	Utilizable wavelength range (cm^−1^)	Penetration depth at 6 μm (μm)	pH range
Diamond	250	45	10	2.4	4000–525	0.70	1–14
GaAs	300	30	6	3.3	10,000–667	0.73	3–12
Silicon	380	30	11	3.4	8900–1500, 475–40	0.70	1–12

### Instrumentation

A Bruker Vertex 70 (Bruker Optics, Ettlingen, Germany) FT-IR spectrometer, equipped with a BioATR II cell (Bruker Optics, Ettlingen, Germany) and a liquid nitrogen (LN_2_)–cooled mercury cadmium telluride (MCT) detector (Bruker Optics, Ettlingen, Germany) were used for comparative studies of the three selected ATR crystal materials. Spectra were recorded at a spectral resolution of 2 cm^−1^, averaging 200 scans each spectrum. Data acquisition was performed with the OPUS 6.5 software package (Bruker Optics, Ettlingen, Germany) and the Essential FT-IR spectroscopy toolbox (Operant LLC, USA). Scanning electron microscopy (SEM) images were acquired with a Helios NanoLab 600 scanning electron microscope (FEI, Eindhoven, The Netherlands). SEM image evaluation was performed with Fiji (distributions of ImageJ) [41]. XPS spectra were recorded with a XPS spectrometer from Physical Electronics (PHI 5800). Chemical structures were drawn with ChemBioDraw (Version 14.0.0.118, Ultra-Package, Perkin Elmer, Waltham, USA).

### Material performance

The BioATR II cell optical setup is based on a two-crystal design with a zinc selenide (ZnSe) focusing crystal and a second, round crystal plate that is in direct contact with the sample. The design of the BioATR II cell enabled application of a high contact pressure between the ZnSe focusing crystal and the IRE, thus ensuring close contact between the two planar crystal surfaces and efficient direct optical coupling. Any use of a fluid in-between the two crystals was deliberately avoided to suppress unwanted absorbance features of such a fluid. Internal reflections within the ATR crystal depend on the thickness and the angle of incidence that are given by the material characteristic refractive index *n*_1_. Homemade laser-cut ATR disks were undoped to maintain transparency in the MIR, since doping drastically reduces the propagation efficiency via free carrier absorbance. Comparative performance tests were conducted with sodium acetate solutions of different concentrations as simple model analyte in aqueous solutions vs. water as background.

### Surface modification and synthesis of gold nanostars

Gold nanostars (AuNSts) were produced via a modified procedure developed by Bibikova et al. [24]. In brief, NSts were fabricated via a modified seed-mediated growth protocol following Yuan et al. [42]. Nanoparticle seeds were prepared using the citrate reduction method introduced by Frens [43, 44]. For AuNSts synthesis, 300 mL of the seed solution was quickly added to 30 μL of 1 M HCl and 10 mL of a 0.25 mM HAuCl_4_ aqueous solution at room temperature under vigorous stirring. In a timely manner, 300 μL of 2 mM AgNO_3_ and 150 mL of 0.1 M AA were added simultaneously to the solution and the reaction solution was stirred for 30 s while its color turned from light red to a grayish blue, indicating successful AuNSt synthesis.

**Fig. 1:**
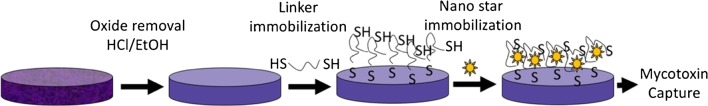
Schematic workflow of the surface modification and subsequent AuNSt immobilization at GaAs ATR disk waveguides

Surface modification of the GaAs ATR crystal was conducted after mounting the precleaned GaAs ATR crystal disks into the BioATR II cell between the ZnSe focusing element and a PTFE sealing ring. Forty microliters auf conc. HCl (32%) was deposited on the GaAs surface for 60 s to remove the native oxide and thus create a clean and active GaAs surface. The HCl solution was subsequently removed by rinsing with about 10 mL dry and degassed ethanol. A small portion of the degassed ethanol was left on the chip surface to suppress regrowth of the native oxide. Forty microliters of a 2 mM α-ω-dimercapto polyethylene glycol (SH-PEG-SH) ethanolic solution (dry and degassed with argon) was added into the sampling tray of the BioATR II cell. The thus prepared chips were immersed for 12 h and subsequently rinsed with approx. 50 mL ethanol to remove unbound physisorbed linker molecules. Afterwards, the chips were immersed for another 12 h in 40 μL of the AuNSt suspension and subsequently rinsed with approx. 50 mL H_2_O, to remove unbound AuNSts from the surface. The surface was dried with a nitrogen stream afterwards. An exemplary workflow is shown in Fig. 1.

### Mycotoxin analysis

Aflatoxin B1 (AFB1) was dissolved in a 2:1 mixture of methanol and chloroform to readily dissolve the AFB1. CHCl_3_ is known to stabilize dissolved AFB1 for shelf storage [45]. Methanol is a commonly utilized solvent for extraction purposes. The binary mixture was chosen to ensure complete dissolution of the AFB1 while keeping the consumption of chlorinated solvents at a moderate level. Acetonitrile, that is routinely used as solvent for mycotoxin standard preparation, was neglected since commercially available acetonitrile often contains relatively high levels of residuals after evaporation which can easily be confused with analyte signals and its non-neglectable toxicity due to metabolization to hydrogen cyanide. For AFB1 detection, 10 μL of solutions with different concentrations was added into the sample tray of the BioATR II cell, with bare and modified (for SEIRAS experiments) GaAs chips mounted. The solvent was left to evaporate, which was complete after about 10 min at room temperature. After experiments, all surfaces were cleaned with sodium hypochlorite to detoxicate AFB1 [46].

## Results

### ATR waveguide comparison

**Fig. 2:**
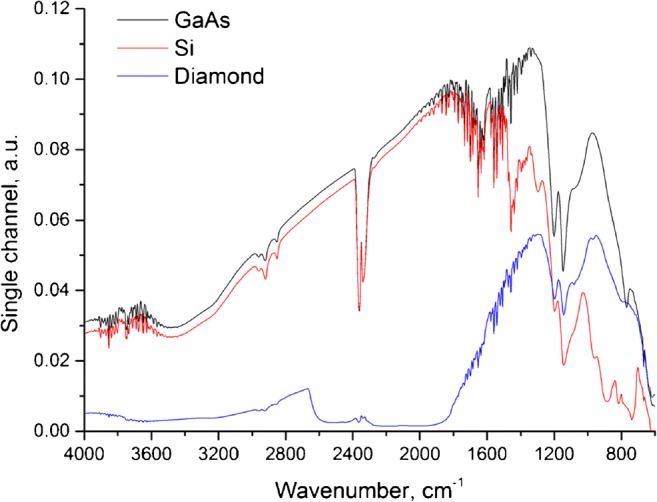
Single-channel spectra of GaAs, Si, and diamond ATR disk waveguides mounted in the same BioATR II cell

Comparing the transmission windows of Si, diamond, and GaAs in the MIR spectral region ranging from 4000 to 600 cm^−1^ indicates that GaAs possesses a transparency window throughout the entire analytically useful MIR spectral window (Fig. 2). Si provides comparable transmission properties at higher wavenumbers, yet is less transparent towards lower wavenumbers. Especially around 1200 cm^−1^, silicon oxide possesses a vibrational mode, and thus reduced transparency in the so-called molecular fingerprint region. Diamond provides high chemical and physical resilience and thus offers versatile options for applications in harsh sensing scenarios including elevated temperatures, strong acids and bases, and contact with abrasive samples. Around 2400 cm^−1^, phonons are excited in the crystal lattice of diamond, which limits the usage of diamond in this spectral regime to wavenumbers lower than 1700 cm^−1^. It should be noted that the single-channel spectra shown in Fig. 2 are superimposed by the device characteristics of the utilized FT-IR spectrometer (i.e., the instrument function), as well as atmospheric water vapor bands around 1600 cm^−1^ and 3700 cm^−1^ and carbon dioxide bands around 2400 cm^−1^. However, since all waveguide materials were studied in the same system and at the same conditions, a fair comparison is ensured.

**Fig. 3:**
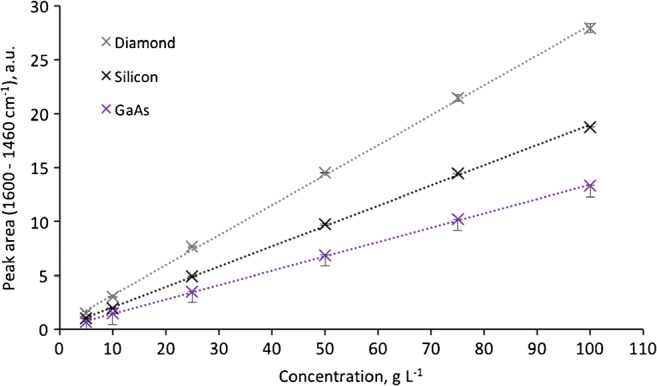
Calibration functions for sodium acetate with ATR disk waveguides made from GaAs, Si, and diamond evaluating the absorbance band from 1600 to 1460 cm^−1^

**Fig. 4:**
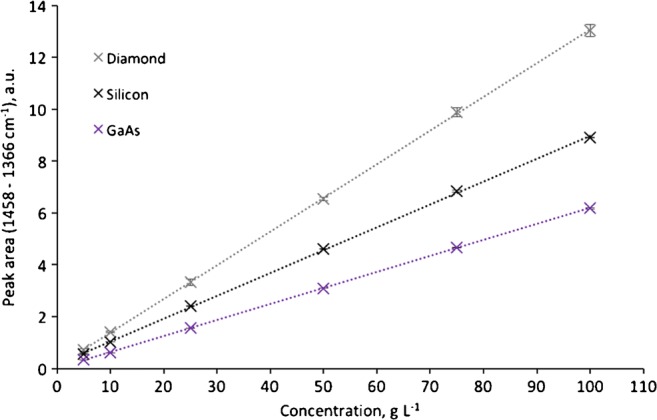
Calibration functions for sodium acetate with ATR disk waveguides made from GaAs, Si, and diamond evaluating the absorbance band from 1458 to 1366 cm^−1^

To directly compare the performance of GaAs to the more routinely used ATR waveguide materials Si and diamond in an ATR-based sensing scheme, sodium acetate was selected as model analyte due to its high solubility in water, its non-toxicity, and its well-pronounced MIR fingerprint spectrum. Calibration functions were established via the integrated peak areas of two different absorbance bands of sodium acetate (Figs. 3 and 4). For the absorption band of the CO_2_ asymmetric stretch centered around 1550 cm^−1^, an integration range from 1600 to 1460 cm^−1^ was used. For the absorption band of the CO_2_^−^ symmetric stretch centered around 1413 cm^−1^, an integration range from 1458 to 1366 cm^−1^ was used. In both cases, diamond shows the steepest slope of the calibration function, which defines the sensitivity followed by Si and GaAs. Due to the wafer disk thickness of 300 μm, the GaAs disks support only 6 internal reflections, i.e., the least number of internal reflections of the three compared disks. Hence, in a next step, the spectral response was normalized to the number of internal reflections by calculating the effectivity *E* of an IRE as *E* = sensitivity/noise, whereas the sensitivity is represented by the slope of the calibration function [47]. Consequently, the energy throughput (Fig. 2) is correlated with the spectral response, and the number of internal reflections is normalized. As shown in Table 2, the resulting normalized efficiency of GaAs ATR waveguides is approx. 40% higher than diamond rendering GaAs a versatile alternative to commonly utilized ATR waveguide materials, if sensitivity is the prime characteristic of interest.

**Table 2:** Evaluation of noise and efficiency of the investigated disk-shaped waveguide materials derived from the calibrations shown in Fig. 3

Peak area:	1600–1460 cm^−1^				
	Slope	Intercept	Standard deviation	Noise	Efficiency
Diamond	0.2784	0.3811	1.1557	0.0018	155
Silicon	0.1883	0.1597	0.7604	0.0020	94
GaAs	0.1328	0.1433	0.7108	0.0006	221

Alternative to calculating the efficiency of the waveguide materials in direct comparison, the LOD as a common analytical figure-of-merit may be derived from the calibration functions shown in Figs. 3 and 4. The limit of detection was calculated according to DIN 32645 taking the *y*-axis intercept, the slope, the standard deviation, and significance level of 96% into account. The *y*-axis intercept has to be considered, as any instrument response due to noise- or non-analyte-related response leads to a potential offset of the calibration function. As summarized in Table 3, all three materials provide LODs in the same concentration range with GaAs being slightly superior substantiating its utility as an alternative IRE waveguide material with similar sensor performance.

**Table 3:** Comparison of the LOD for aqueous solutions of sodium acetate for two different absorbance bands using ATR waveguide disks made from GaAs, Si, and diamond

	LOD (g L^−1^; × 1000 ppm)	
	1600–1460 cm^−1^	1458–1366 cm^−1^
Diamond	3.4	2.8
Silicon	2.2	1.8
GaAs	2.1	0.8

### GaAs surface modification and nanoparticle immobilization

As a macroscopic tool to investigate surface modification procedures, water contact angle measurements were performed at different stages of the GaAs modification process. Etched GaAs showed a contact angle of 61° ± 7° indicating a quite hydrophobic surface due to removal of surface oxide and hydroxide functional groups, as confirmed in literature [10]. The HS-PEG-SH functionalized GaAs surfaces lead to a contact angle of 19° ± 5°. These results are also in accordance with literature values for the functionalization of surfaces with SAMs via organo-dithioles, which are hydrophilic due to formation of hydrogen bonds with water molecules [48].

**Fig. 5:**
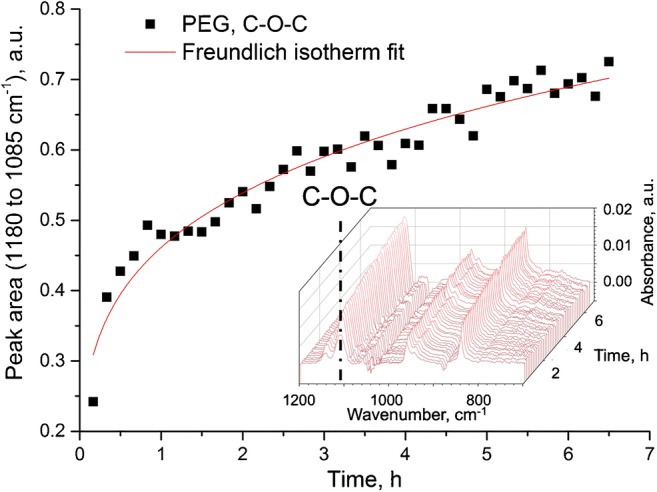
IR spectral tracking of the C-O-C band of the HS-PEG-SH polymer during immobilization at the GaAs waveguide disk surface during the first 7 h of the immobilization. The Freundlich isotherm–like fit follows a relation according to *y* = 0.46·*x*^1/4.46^. The inset shows the time-resolved development of the IR spectra with the C-O-C band highlighted by a dashed line

GaAs—as an ATR waveguide—enables direct in situ monitoring of surface modification protocols. To study the immobilization kinetics at GaAs in more detail, MIR spectra were recorded during the immobilization process of HS-PEG-SH at the deoxidized surface in situ via IR difference spectra. A background spectrum was recorded after depositing 40 μL of the HS-PEG-SH solution in ethanol at the waveguide surface and closing the sampling chamber lid. Sample spectra were recorded subsequently after 10 min each for 7 h. The C-O-C band of the HS-PEG-SH molecule was selected as the exemplary band and evaluated during the modification process at GaAs surface over time. For this purpose, the absorption band of the C-O stretch vibration of the C-O-C ether functional group centered around 1134 cm^−1^ was integrated from 1180 to 1085 cm^−1^. As shown in Fig. 5, the band representing the C-O-C vibration strongly increases during the first hour of immersion, and afterwards increases according to a Freundlich isotherm–like behavior. Adsorption kinetics according to the Freundlich model assume a decreasing enthalpy of adsorption due to an increased surface coverage, and thus, non-equal adsorption places due to repulsion of the adsorbed molecules. Such a behavior may indeed be expected from the chemical and structural composition of the large HS-PEG-SH molecules. Furthermore, complete surface coverage cannot be modeled with a Freundlich isotherm, which is in further accordance with the expected behavior of the HS-PEG-SH adsorption kinetics.

Due to the length of the PEG back chain, potential bilayers or otherwise physisorbed HS-PEG-SH molecules within the chemisorbed and strongly bound molecules are expected in close vicinity of the surface, and therefore within the penetration depth of the evanescent field. Hence, sufficient and thorough solvent rinsing after the immobilization procedure has to be ensured for removing non-covalently bound molecules at the waveguide surface.

**Fig. 6:**
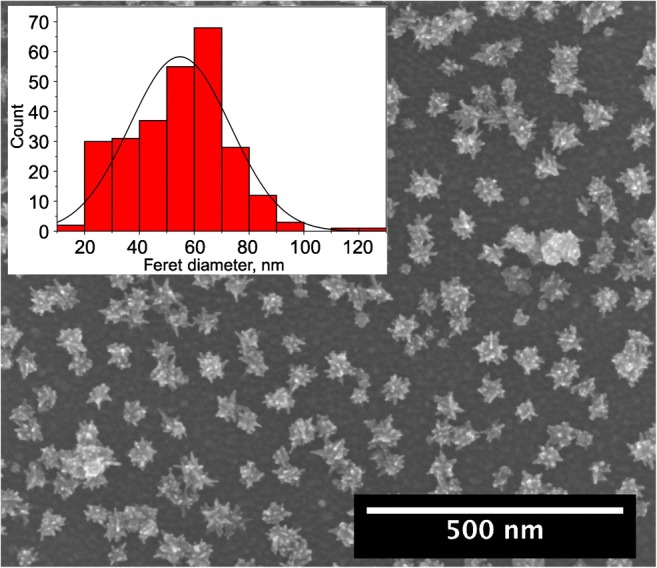
Scanning electron microscope (SEM) image of a representative section of the immobilized AuNSts at the GaAs waveguide disk surface with an average feret diameter of 55 nm of the AuNSts

To further evaluate the efficiency of the immobilization and AuNSt binding performance, SEM images and XPS spectra were recorded at the finally modified waveguide surface. The exemplary SEM image shown in Fig. 6 readily shows immobilized AuNSts at the HS-PEG-SH-modified surface after thorough rinsing with water and blow drying. Thus, the prepared AuNSts show a narrow size distribution at an average feret diameter of 55 ± 30 nm, which results from the seed-mediated approach [24]. Furthermore, the AuNSt surface coverage is calculated at approx. 30%, as derived from a representative area of approx. 1.5 μm^2^. This uniformity is of importance to ensure reproducible surface coverage and consequently, reliable sensor response during repeated experiments.

**Fig. 7:**
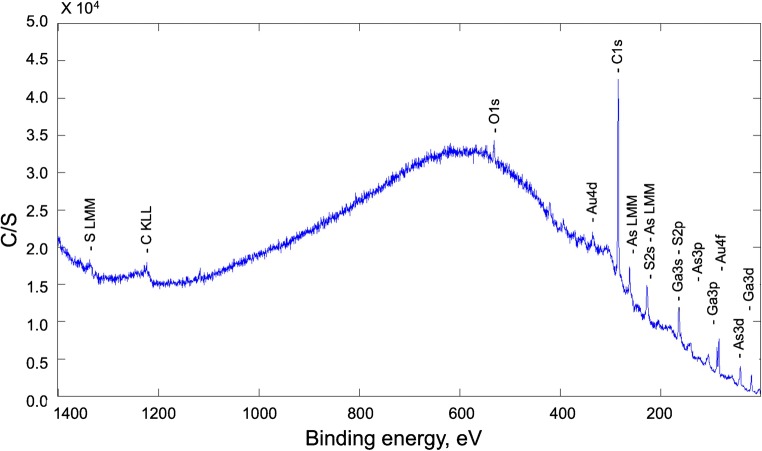
XPS spectrum of a HS-PEG-SH-modified GaAs waveguide disk with immobilized AuNSts

Elemental surface coverage was evaluated via XPS studies addressing the atomic concentration at approx. 1–12 nm depth from the sample surface. An exemplary XP spectrum is shown in Fig. 7, and the derived atomic surface concentrations are summarized in Table 4.

**Table 4:** Atomic surface concentration derived from the XPS analysis (*N* = 2)

Atom peak	C1s	O1s	S2p	Ga3d	As3d	Au4f
Atomic concentration (%)	73	4	11	6	5	1

C, O, S, and Au introduced by modifying a GaAs surface with thiol-modified polyethylene glycol and subsequent gold nanostar immobilization are present in the XP spectra. The dominating atomic concentration of carbon of about 73% indicates possible shielding of the generated radiation from the other elements, since a ratio of approx. 2:1 for C:O correlates to the PEG component.

### Direct broadband mycotoxin detection via SEIRA on GaAs waveguide disks

Due to the signal amplifying SEIRA effect, low-level detection of relevant analytes may benefit in addition to chem/biorecognition schemes from such signal enhancement strategies. Herein, the detection of trace levels of ABF1 was selected serving as a relevant candidate to evaluate potential benefits of HS-PEG-SH-modified ATR waveguide surfaces decorated in addition with AuNSts.

**Fig. 8:**
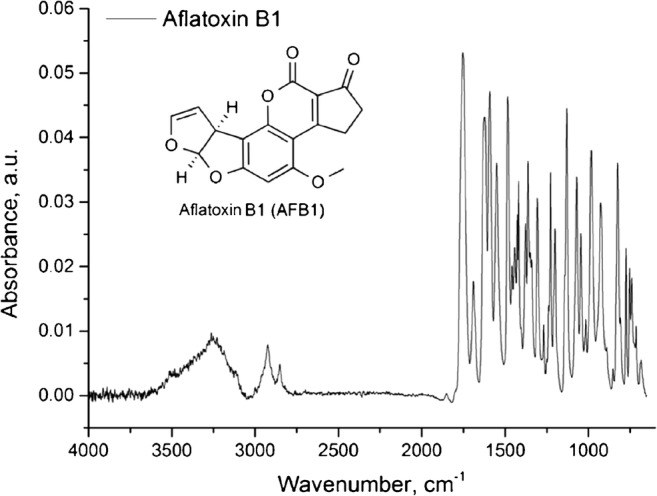
IR-ATR spectrum of 600 ppm AFB1 after solvent evaporation (2 cm^−1^ spectral resolution; 200 scans averaged per spectrum)

An exemplary IR-ATR spectrum of 600 ppm AFB1 after solvent evaporation is shown in Fig. 8. The IR fingerprint shows the anticipated complex absorbance pattern associated with large organic molecules comprising a variety of functional moieties, as present in AFB1. As GaAs is widely transparent in the fingerprint region, identification of pure (bio)molecules via unique MIR fingerprint spectra is readily achievable.

**Fig. 9:**
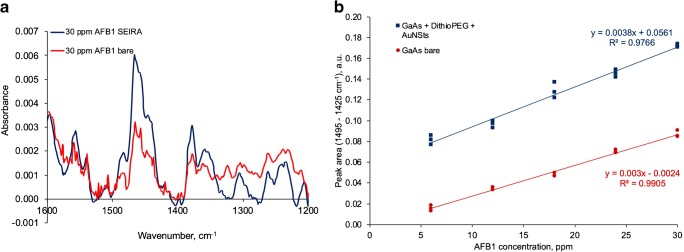
**a** Raw spectra of AFB1 fingerprint spectra with a concentration of 30 ppm on a bare substrate (red line) and on a modified SEIRA-active substrate (blue line). **b** Calibration functions of the spectral response of an exemplary AFB1 band integrated from 1495 to 1425 cm^−1^ at a bare (red) vs. a AuNSt-modified GaAs waveguide disk surface (blue) (*N* = 3)

Comparative calibrations for the trace-level detection of AFB1 in the low ppm concentration regime with and without SEIRA-active substrate are shown in Fig. 9. To avoid interference with absorption bands of atmospheric water vapor, integration of an absorption band ranging from 1495 to 1425 cm^−1^ was selected to represent the AFB1 concentration. The SEIRA-active substrate shows an analytical signal amplification by a factor of approx. 4 vs. the non-enhanced counterpart. This enhancement factor is similar to previously reported enhancement factors for biomolecules [49]. This enhancement factor is clearly at the lower range of possible SEIRA enhancement factors reported in literature, where enhancement factors up to 1000 have been shown [50]. Potential strategies towards higher enhancement factors are increasing the surface coverage density of AuNSts [5]. The SEIRA calibration function shows a slight increase in slope, i.e., sensitivity by approx. 20%. However, the corresponding standard deviations of the SEIRA-based calibration function appear elevated as well. This increased sample-to-sample variance may be attributed to minor differences between individual modified IRE surfaces.

Deriving the LOD of AFB1 at bare GaAs according to DIN 32645, a value 4000 ppb has been calculated. For the AuNSt-decorated substrate, a slightly improved LOD of 3500 ppb was resultant. The presented exemplary surface decoration motif led to an LOD enhancement of approx. 14%. This improvement may be sufficient when working at regulatory limits for selected analytes boosting the performance by an order of magnitude into a useful range. As an alternative or in addition, incorporating more sophisticated nanostructures giving rise to SEIRAS effects along with selective analyte capture and enrichment schemes is anticipated to further enhance future sensor performance. Likewise, more sophisticated chemometric strategies need to be applied in combination with such motifs to extract relevant analyte signals from more complex real-world analyte samples.

## Conclusions and outlook

The implementation of GaAs as an internal total reflection waveguide for broadband IR ATR sensing concepts has been evaluated in detail and has been confirmed as a versatile alternative to conventionally used ATR waveguide materials such as Si or diamond. Similar to gold surfaces, GaAs semiconductors facilitate surface chemistries via covalent thiol bonds, thus enabling a wide variety of immobilization architectures based on this inherently robust surface modification towards chem/biosensing interfaces. GaAs is a widely used semiconductor platform for thin-film on-chip waveguide architectures in photonic circuitries. Herein, we evaluate the utility of GaAs serving as an active sensing element along with potential surface modification strategies for the implementation of highly integrated photonic chem/biosensors at a chip scale based on a new semiconductor platform. As a result, IR light sources and detectors operating in the MIR that are based on GaAs architectures may now be fully integrated with chem/biodetection schemes using the same material as the optical transducer element facilitating evanescent field sensing. Using surface-enhanced infrared effects enables the additional amplification of the obtained analytical signals. While thin-film waveguide technology based on GaAs architectures has already demonstrated its utility, broadband direct spectroscopies may likewise benefit from advanced ATR waveguide designs and innovative waveguide materials [51, 52].
